# Scorpions are taking over: the silent and escalating public health crisis in Brazil

**DOI:** 10.3389/fpubh.2025.1573767

**Published:** 2025-05-08

**Authors:** Manuela B. Pucca, Joeliton S. Cavalcante, Sewbert R. Jati, Felipe A. Cerni, Rui Seabra Ferreira, Eliane C. Arantes

**Affiliations:** ^1^Department of Clinical Analysis, School of Pharmaceutical Sciences, São Paulo State University (UNESP), Araraquara, SP, Brazil; ^2^Center for the Study of Venoms and Venomous Animals of UNESP (CEVAP), São Paulo State University (UNESP), Botucatu, São Paulo, Brazil; ^3^Graduate Program in Tropical Medicine (PPGMT), State University of Amazonas, Manaus, Brazil; ^4^Medical School, Federal University of Roraima (UFRR), Boa Vista, Roraima, Brazil; ^5^Graduate Program in Tropical Diseases, Botucatu Medical School (FMB), São Paulo State University (UNESP), Botucatu, São Paulo, Brazil; ^6^Department of Biomolecular Sciences, School of Pharmaceutical Sciences of Ribeirão Preto, University of São Paulo, Ribeirão Preto, Brazil

**Keywords:** scorpion, scorpionism, Brazil, *Tityus serrulatus*, envenoming, venomous animal

Scorpionism, or scorpion sting envenoming, is a global issue (1), affecting several regions worldwide, including north-Saharan Africa, Sahelian Africa, South Africa, Near and Middle-East, South India, Mexico and Latin America (2–6). In the Americas, Brazil, Paraguay, Bolívia, Mexico, Guyanas and Venezuela have witnessed a particularly alarming rise in scorpionism over recent decades, evolving into a significant public health crisis (1, 6–8). This surge is driven by a complex interplay of environmental, social, and biological factors (9). Rapid, unplanned urbanization, especially in areas with poor infrastructure, inadequate sanitation, and inconsistent waste management, creates ideal environments for scorpions to thrive (10). These conditions provide abundant shelter in debris, sewage systems, and within homes, bringing humans and scorpions into close proximity. Additionally, climate change, marked by hotter summers and alternating periods of intense rainfall and drought, further facilitates the proliferation of scorpion populations, as these creatures are highly adapted to warm and humid environments (11).

In Brazil, the genus *Tityus* is medically significant, with *T. serrulatus, T. bahiensis, T. stigmurus*, and *T. obscurus* capable of causing clinically significant envenomation (12). The clinical manifestation including: (i) local manifestations: pain, burning sensation, erythema, paresthesia, swelling, and tingling; (ii) minor systemic manifestations: agitation, headache, nausea, vomiting, sweating, unhealthy pallor, salivation, somnolence/lethargy, tachycardia, hypertension, hypothermia, hyperthermia, myoclonia, fasciculation, ataxia, dystonia, miosis, and mydriasis; and (iii) major systemic manifestations: hypotension, ventricular arrhythmia, bradycardia, cardiovascular collapse, cyanosis, dyspnea, pulmonary edema, paralysis, and Glasgow score < 6 (in absence of sedation) (13–15). Laboratory abnormalities include hyperglycemia, hypokalemia, leukocytosis, elevated CK, CK-MB, and troponin T levels in serum, bicarbonate consumption, and increased base deficit and blood lactate (16–18). In cases of minor and major systemic manifestations, associated or not with laboratory alterations, treatment with antivenom is indicated.

Children and the older adults are especially vulnerable due to their reduced capacity to withstand the rapid and overwhelming effects of the venom (19). Given the potency and swift action of the venom, immediate medical attention is critical, often requiring the administration of antivenom and intensive care. This deadly venom cocktail, combined with the species' adaptability and rapid reproductive rate, firmly establishes *Tityus* scorpions as a major public health concern in Brazil.

**Figure 1:**
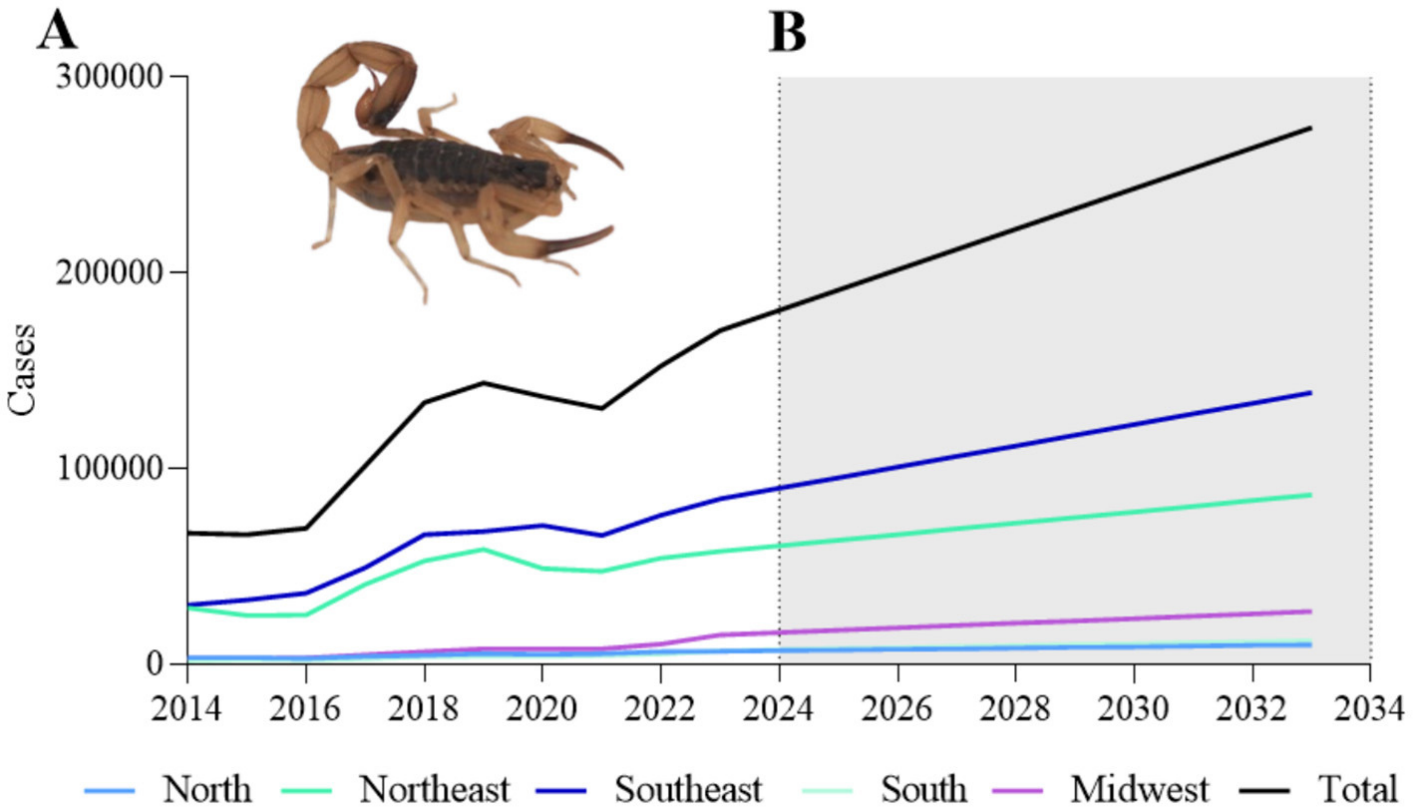
Reported and projected cases of scorpionism in Brazil. **(A)** Historical data on scorpionism cases (2014–2023). Data obtained from SINAN (Sistema de Informação de Agravos de Notificação, *i.e*., the Brazilian Notifiable Diseases Information System), January 2024. **(B)** Projected (2024–2033) based on trend analysis. The black line represents the total number of cases, while the colored lines indicate different Brazilian regions, with improved color contrast to distinguish geographical differences more effectively. The shaded area denotes the projected period. A photograph of *Tityus serrulatus* (credited to Felipe A. Cerni) is included, highlighting this species as the primary cause of scorpionism cases in Brazil.

In Brazil, 1,171,846 cases were reported between 2014 and 2023. The Southeast region was the most affected during this period, with 580,013 cases (49.5%), followed by the Northeast region, with 439,033 cases (37.5%). Over time, we have seen an increase in cases annually, with a decrease between 2020 and 2021 caused by the trajectory of the COVID-19 pandemic. During that period, isolation measures, hospital overload, and fear of contamination by victims may have generated low rates of scorpion stings. After the pandemic, the number of new cases of scorpionism increased from 136,795 and 130,665 cases in 2020 and 2021, respectively, to 152,384 in 2022, reaching 170,616 cases in 2023 (Figure 1A). It is noteworthy that, compared to 2014, 2023 showed a 254.70% increase in reported cases, jumping from 66,986 to 170,616 cases (data from 2024 were updated and available during the research). In this scenario, we performed a projection using the ARIMA (Autoregressive Integrated Moving Average) model (20) that regulates time series with trends, also known as Box-Jenkins procedure (21). Based on error metrics such as RMSE (Root Mean Square Error), a trendline was used to project the next 10 years based on historical data and its variation by region of Brazil. The data obtained indicate that between 2024 and 2033, ~274,246 new cases of scorpionism may occur. Of these, 2,148,576 cases in the Southeast, 182,836 in the South, 1,383,800 in the Northeast, 158,573 in the North, and 404,219 in the Central-West (Figure 1B). In total, we expect 2,095,146 new cases to occur between 2025 and 2033 (Table 1).

**Table 1:** Number of reported and projected cases of scorpionism in Brazil by region (2014–2033).

**Year**	**North**	**Northeast**	**Southeast**	**South**	**Midwest**	**Total**
2014	3,332	28,776	30,233	1,738	2,907	66,986
2015	3,324	24,882	32,855	2,323	2,736	66,120
2016	2,948	25,022	36,297	2,034	3,064	69,365
2017	4,004	40,756	49,312	2,660	4,705	101,437
2018	4,561	52,764	66,184	3,779	6,405	133,693
2019	5,427	58,633	67,794	4,043	7,888	143,785
2020	5,056	48,922	70,962	4,062	7,793	136,795
2021	5,356	47,455	65,719	4,353	7,782	130,665
2022	6,482	54,183	76,143	5,281	10,295	152,384
2023	6,617	57,640	84,514	6,904	14,941	170,616
2024	6,946	60,526.4	89,942.1	7,420.6	16,144.4	180,979
2025	7,274	63,412.8	95,370.2	7,937.2	17,347.8	191,342
2026	7,603	66,299.2	10,0798.3	8,453.8	18,551.2	201,705
2027	7,931	69,185.6	10,6226.4	8,970.4	19,754.6	212,068
2028	8,260	72,072	11,1654.5	9,487	20,958	222,431
2029	8,588	74,958.4	11,7082.6	10,003.6	22,161.4	232,794
2030	8,917	77,844.8	12,2510.7	10,520.2	23,364.8	243,157
2031	9,245	80,731.2	12,7938.8	11,036.8	24,568.2	253,520
2032	9,574	83,617.6	13,3366.9	11,553.4	25,771.6	263,883
2033	9,902	86,504	138,795	12,070	26,975	274,246
Mean	328.5	2,886.4	5,428.1	516.6	1,203.4	10,363
Increase	1.5	1.5	1.6	1.7	1.8	8.1

Reported data (2014–2023) were obtained from SINAN SINAN (Sistema de Informação de Agravos de Notificação, i.e., the Brazilian Notifiable Diseases Information System), January 2024. In gray, the projected data.

The dramatic rise in scorpionism is placing immense pressure on Brazil's public health system. Urbanization has made encounters with scorpions increasingly common, largely due to the accumulation of garbage and inadequate sanitation, which create favorable habitats with abundant resources for these arachnids (10, 22, 23). Additionally, their proliferation is facilitated by parthenogenesis (24), as observed in *Tityus serrulatus*, a species composed exclusively of females. Given that these scorpions can survive for extended periods—up to 400 days—without food (25), these factors contribute to a rising population density in urban areas, significantly increasing the risk of scorpionism.

If the numbers are already so alarming, what happens when we consider the vast underreporting of scorpionism? The real scale of this issue is likely far greater than the recorded statistics suggest. While pediatric cases requiring medical intervention represent only a small fraction of the affected population, adult cases, often dismissed as minor due to localized pain that can be managed with analgesics, frequently go unreported. Many victims choose to treat themselves at home or forego treatment entirely, allowing the true extent of scorpionism to remain hidden. Thus, this underreporting is not just a statistical issue; it is a major obstacle to effective control measures. Without accurate data, health authorities struggle to assess the real burden of scorpionism and implement targeted interventions.

The official numbers paint a deeply concerning picture, but the reality is likely far worse. Scorpionism is not just an emerging public health issue—it is a hidden epidemic growing unchecked. Worsened by poor sanitation, rapid urban expansion, and widespread public unawareness, this silent crisis continues to escalate, placing an increasing number of lives at risk.

This data clearly illustrates the alarming rise in scorpionism cases over the years. From 2014 to 2023, cases surged by more than 250%, and projections indicate a continued upward trend, with an estimated 274,246 cases by 2033—an increase of 60.7% compared to 2023. Notably, all regions of Brazil are experiencing this surge, with an annual average increase of 10,363 cases, reflecting a rapidly expanding public health crisis.

Given the relentless rise in scorpionism and the significant challenges posed by underreporting, immediate action is essential to prevent further escalation. Strengthening public awareness campaigns on scorpion risks, preventive measures, and the importance of case reporting is crucial for obtaining more accurate data and implementing effective control strategies. Without decisive intervention, scorpionism will continue its upward trajectory, placing an even greater burden on Brazil's healthcare system and putting public safety at increasing risk.
